# Eye-tracking during simulation-based echocardiography: a feasibility study

**DOI:** 10.1186/s12909-023-04458-z

**Published:** 2023-07-01

**Authors:** Christina Hafner, Vincenz Scharner, Martina Hermann, Philipp Metelka, Benedikt Hurch, Daniel Alexander Klaus, Wolfgang Schaubmayr, Michael Wagner, Andreas Gleiss, Harald Willschke, Thomas Hamp

**Affiliations:** 1 Department of Anaesthesia, General Intensive Care and Pain Medicine, Medical University of Vienna, Spitalgasse 23, 1090 Vienna, Austria; 2 Ludwig Boltzmann Institute Digital Health and Patient Safety, Vienna, Austria; 3 Department of Pediatrics, Comprehensive Center for Pediatrics, Medical University of Vienna, Vienna, Austria; 4 Center for Medical Statistics, Informatics, and Intelligent Systems, Medical University of Vienna, Vienna, Austria; 5 Emergency Medical Service Vienna, Radetzkystraße 1, 1030 Vienna, Austria

**Keywords:** Echocardiography, Education, Eye-tracking

## Abstract

**Introduction:**

Due to the technical progress point-of-care ultrasound (POCUS) is increasingly used in critical care medicine. However, optimal training strategies and support for novices have not been thoroughly researched so far. Eye-tracking, which offers insights into the gaze behavior of experts may be a useful tool for better understanding. The aim of this study was to investigate the technical feasibility and usability of eye-tracking during echocardiography as well as to analyze differences of gaze patterns between experts and non-experts.

**Methods:**

Nine experts in echocardiography and six non-experts were equipped with eye-tracking glasses (Tobii, Stockholm, Sweden), while performing six medical cases on a simulator. For each view case specific areas of interests (AOI) were defined by the first three experts depending on the underlying pathology. Technical feasibility, participants’ subjective experience on the usability of the eye-tracking glasses as well as the differences of relative dwell time (focus) inside the areas of interest (AOI) between six experts and six non-experts were evaluated.

**Results:**

Technical feasibility of eye-tracking during echocardiography was achieved with an accordance of 96% between the visual area orally described by participants and the area marked by the glasses. Experts had longer relative dwell time in the case specific AOI (50.6% versus 38.4%, *p* = 0.072) and performed ultrasound examinations faster (138 s versus 227 s, *p* = 0.068). Furthermore, experts fixated earlier in the AOI (5 s versus 10 s, *p* = 0.033).

**Conclusion:**

This feasibility study demonstrates that eye-tracking can be used to analyze experts and non-experts gaze patterns during POCUS. Although, in this study the experts had a longer fixation time in the defined AOIs compared to non-experts, further studies are needed to investigate if eye-tracking could improve teaching of POCUS.

**Supplementary Information:**

The online version contains supplementary material available at 10.1186/s12909-023-04458-z.

## Introduction

Eye-Tracking is an objective tool, which delivers insights into gaze behavior of the operator during medical procedures [1–3]. With this innovative technology the visual focus of a healthcare provider during performance of medical tasks can be determined [4–6]. Although gaze behavior cannot be equated with cognition, it enables a better understanding of the thought processes of operators with various levels of expertise. Thus, differences in gaze patterns between experts and novices have been reported in several studies. Experts mainly remain longer in predefined areas of interest and have a clear focus to their gaze [7–9]. Moreover, studying an expert’s gaze behavior may even play a role in medical education for better understanding an expert’s approach and method, especially for challenging tasks (e.g., ultrasonography in critical care medicine).

Due to the technical progress of portable ultrasound devices and appropriate education of nonradiologists and noncardiologists in sonography, point-of-care ultrasound (POCUS) is increasingly used in critical care medicine [10]. POCUS provides real-time diagnosis and supports physicians in decision making to develop treatment strategies [11]. As clinicians with insufficient knowledge may harm patients with inaccurate diagnosis, training to acquire adequate competence is essential. However, the optimal training (e.g., course design, practical training) for becoming competent and the best way in which experts can deliver support for novices during POCUS is unknown.

A handful studies investigated the technology of eye-tracking in medical education for training, assessment as well as a feedback tool [4, 8, 12, 13]. Several studies reported that gaze trained novices demonstrated patterns closer to experts [12, 14–16]. Furthermore, in the educational setting eye-tracking can even be used as a feedback tool. This allows trainees to focus on the area, where training and improvement is required [12]. Due to these findings, insights into gaze behavior between experts and novices may be a useful step for teaching POCUS. Nevertheless, eye-tracking has not been studied during echocardiography.

Therefore, the aims of this study were to investigate technical feasibility as well as usability of eye-tracking during simulation-based echocardiography and to evaluate differences of gaze patterns between experts and non-experts.

## Methods

### Study design

This feasibility study was approved by the data protection committee of the Medical University of Vienna, Austria (05/2021) and was performed according to the Declaration of Helsinki guidelines regarding research on human subjects. Due to the manikin-based simulation character of this study the ethics board gave this study an exempt status. This study was performed between September 2021 and March 2022 at the Medical University of Vienna.

### Participants

In this study, nine clinicians with expertise on critical care echocardiography and six residents with limited expertise were recruited to voluntarily participate in the study. Informed written consent was obtained from all participants before beginning. Experts were specialists from the Department of Internal Medicine as well as from the Department of Anaesthesia, Intensive Care Medicine and Pain Medicine with diploma and regular use of focus assessed transthoracic echocardiography (FATE) for more than two years. As non-experts, six residents of the Department of Anaesthesia, Intensive Care and Pain Medicine with theoretical courses, but low practical expertise on echocardiography, were included. All residents participated in an 1 h online repetition on the basics of echocardiography prior to the study on the basics of echocardiography.

### Experimental setup

Prior to the study initiation, six pre-installed medical cases of the simulator U/S MENTOR™ (3D Systems, Airport City, Israel) were selected by the study team. For each, scenario participants were given details about history and clinical symptoms of the patient (supplementary data 1). To ensure reproducibility and to offer optimal conditions for the interpretation of ultrasound images, a standardized set-up for simulator, ultrasound monitor, chair and ambient lighting was provided and checked before each scenario. Participants were equipped with mobile Tobii Pro 2.0 eye-tracking glasses (Tobii Pro, Stockholm, Sweden). Calibration of the eye-tracking glasses was performed according to the manufacturer’s instruction before each scenario. During echocardiography, the participants described orally at which anatomical structure they were looking at. Audio and eye movements at a rate of 50 Hz were recorded with the eye-tracking glasses. For analysis, the Tobii Pro Glasses Analyzer Software (Tobii Pro, Stockholm, Sweden) was used. The areas of interests (AOIs) were determined by the study team for each of the standard FATE views (parasternal long axis, parasternal short axis, apical and subxiphoidal) for every scenario in accordance with the fixation patterns observed from the first three experts. The sequence of the scenarios was randomized using latin squares to account for habituation and learning effects.

### Outcome measures

Feasibility was defined as the accordance of the orally described area of visual attention with the area of attention marked by the eye-tracking glasses (*n* = 12). For differences between experts (*n* = 6) and non-experts (*n* = 6), the relative dwell time (focus on a specific AOI) as well as the absolute time to first fixation in the case specific AOI, and total duration of echocardiography were assessed. The usability of the eye-tracking glasses was evaluated by all participants (*n* = 15) after the last scenario using a questionnaire with 11 items about comfort and distraction (5-point Likert scale for each item) which was also used in a previous feasibility study [17]. Baseline characteristics included age, gender, medical background, years of professional experience as well as years of experience in echocardiography.

### Sample size calculation

For this feasibility study, sample size was based on previous work by Borg et al., who analyzed the feasibility of eye-tracking glasses during ultrasound-guided regional anaesthesia with 5 experts and 5 non-experts [6]. For determination of the case specific AOIs it was suggested that in total 3 experts were sufficient.

### Statistical analysis

Descriptive statistical analysis was performed using SPSS Version 27 (IBM, Armonk, NY, USA), while feasibility, usability, and the comparison between experts and non-experts was analyzed using SAS 9.4 (SAS Institute, Cary, NC, USA, 2016). Demographic data are presented as median (minimum and maximum) due to an asymmetric distribution of some variables. The accordance of the verbally stated area of visual attention with the marked area are reported as percentage of the correctly stated areas of the total number of verbalized areas of visual attention. Relative dwell time, as well as first time of fixation and duration of echocardiography, are described as median (minimum and maximum) for each group (experts; non-experts). Differences between these groups were estimated using a linear mixed model with fixed effects group, case, and period, and a random proband effect to adjust for period effects and dependence within probands. The conditional model residuals were successfully checked for approximate normal distribution and for outliers and influential observations. The conditional R-squared measure is calculated using the function r.squaredGLMM in the R package ‘MuMln’ Version 4.3.0 [18]. Time to first fixation was log-transformed to stabilize the residual distribution (group differences are back-transformed to result in a relative group difference); an additional random effect for view within proband was added. Usability of eye-tracking glasses are reported as mean for each group.

## Results

### Participants’ characteristics

Demographic data of the participants are depicted in Table 1.

**Table 1:** Demographic data

	**AOI experts (*n* = 3)**	**experts (*n* = 6)**	**non-experts (*n* = 6)**
**Age in years** median (min – max)	52 (34–60)	38 (26–46)	33 (29–34)
**Male** (n (%))	3 (100%)	4 (67%)	4 (67%)
**Medical Background**			
Cardiology (n (%))	3 (100%)	3 (50%)	0 (0%)
Anaesthesia (n (%))	0 (0%)	3 (50%)	6 (100%)
**Professional experience in years** median (min – max)	20 (9–30)	10 (7–19)	6 (4–9)
**Echocardiography experience in years** median (min – max)	11 (9–15)	8.5 (2–12)	5 (0.5–7)

Data are presented as median (minimum and maximum) and numbers (%)

### Technical feasibility

During the entire duration of all 72 recordings, the area of attention was marked by the eye-tracking glasses. In total 1366 areas were orally explained. In 96% of these, the orally described area was in accordance with the marked area of attention.

### Differences between experts and non-experts

**Fig. 1:**
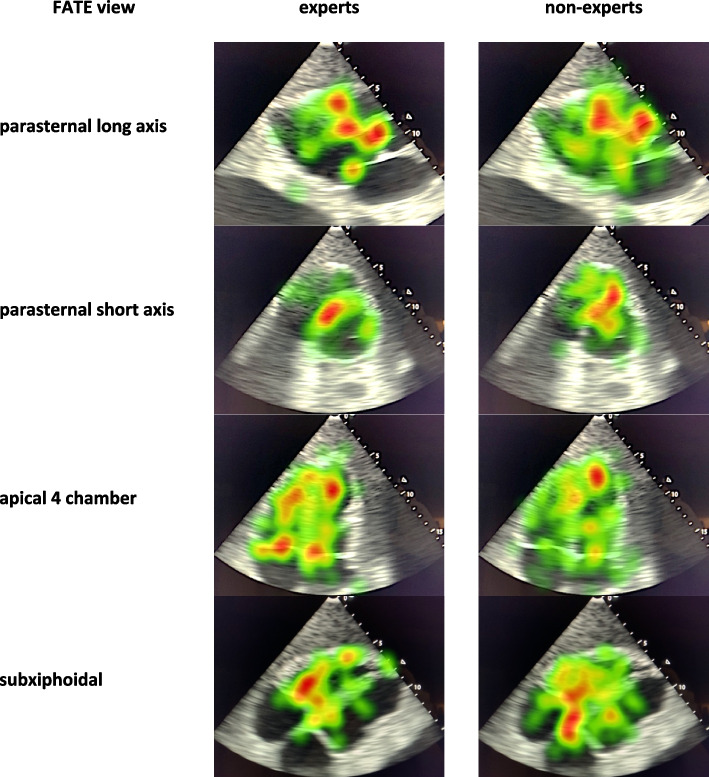
Heatmap of gaze patterns (case 2 pulmonary embolism) between experts (*n* = 6) and non-experts (*n* = 6). Intensity of redness correlates with the dwell time in the area

Differences between experts and non-experts are depicted in Table 2. Although not statistically significant, experts had a longer relative dwell time in the defined AOI than non-experts (raw data across all cases: 50.6% versus 38.4%; adjusted difference: 9.4%-points, 95% confidence interval (CI): -0.9 to + 19.6, *p* = 0.072, R^2^ 0.51). Furthermore, experts performed a faster ultrasound examination (median raw data across all cases:138 s versus 227 s; adjusted difference: 68 s, 95% CI: -5 to + 142, *p* = 0.068, R^2^ 0.72). The median time to first fixation in the AOI (supplementary data 2) was significantly lower in the expert group compared to the non-expert group (median raw data across all cases and views: 5 s versus 10 s; adjusted relative difference: 45% reduced fixation time, 95% CI: 68% to 5%, *p* = 0.033, R^2^ 0.18). Differences of gaze patterns between experts and non-experts are demonstrated in Fig. 1.

**Table 2:** Differences between experts (*n* = 6) and non-experts (*n* = 6)

	**Relative dwell time in the AOI** median (min – max)	**Duration of echocardiography** median (min – max)
**Case 1 (pericardial effusion)**		
experts	40 (20–61) %	127 (88–427) sec
non-experts	34 (18–51) %	184 (100–269) sec
**Case 2 (pulmonary embolism)**		
experts	41 (29–67) %	149 (70–235) sec
non-experts	35 (24–49) %	223 (117–360) sec
**Case 3 (acute myocardial infarction)**		
experts	56 (39–71) %	159 (100–353) sec
non-experts	35 (27–58) %	234 (183–341) sec
**Case 4 (cardiac myxoma)**		
experts	59 (30–71) %	130 (88–427) sec
non-experts	55 (34–77) %	232 (159–303) sec
**Case 5 (bicuspid aortic valve)**		
experts	49 (24–76) %	145 (88–226) sec
non-experts	32 (24–48) %	295 (152–414) sec
**Case 6 (left ventricular pseudo-aneurysm)**		
experts	59 (40–76) %	131 (77–251) sec
non-experts	41 (28–65) %	193 (151–216) sec
**Total**		
experts	50.6 (30–70) %	138 (85–320) sec
non-experts	38.4 (26–58) %	227 (144–316) sec

Data are presented as median (minimum and maximum)

### Usability of eye-tracking glasses

At the end of all 6 scenarios each participant evaluated the subjective experience with the eye-tracking glasses (Table 3). 13 of 15 participants denied that eye-tracking glasses distracted them from performing echocardiography. Most participants disagreed with discomfort due to eye-tracking glasses. Whereas all non-experts would wear eye-tracking glasses during a real scenario, only 2 of 9 experts considered this option.

**Table 3:** Usability of eye-tracking glasses

	**AOI experts (*n* = 3)** median (min–max)	**experts (*n* = 6)** median (min–max)	**non-experts (*n* = 6)** median (min–max)
I would wear eye tracking glasses during a real scenario	3 (1–3)	2.5 (1–4)	5 (3–5)
During the scenario, I forgot that I was wearing the glasses	3 (2–5)	4 (1–5)	4 (2–5)
Impaired my visual field in performing echocardiography	1 (1–2)	1 (1–1)	1.5 (1–3)
Slid down on my nose	1 (1–4)	1 (1–4)	1.5 (1–4)
Felt uncomfortable for my eyes	2 (1–3)	1 (1–3)	2 (1–3)
Felt uncomfortable for my ears	2 (2–3)	1 (1–1)	1 (1–4)
Feld uncomfortable on my nose	3 (2–4)	1 (1–5)	1.5 (1–3)
Distracted me during the whole scenario	1 (1–2)	1 (1–5)	1 (1–2)
Distracted me because of the wire	1 (1–2)	1 (1–2)	1 (1–2)
Distracted me from performing echocardiography	1 (1–2)	1 (1–4)	1 (1–2)

Usability of eye-tracking was evaluated using a 5-point likert scale (1 disagree to 5 agree). Data are presented as median and interquartile range

## Discussion

To our knowledge, this is the first study evaluating eye-tracking during simulation-based echocardiography. The findings of this pilot study demonstrated that eye-tracking during transthoracic echocardiography was technically feasible, relatively comfortable for the performer, and suggested that non-experts and experts have different gaze patterns.

Eye-tracking and assessment of gaze behavior holds a great potential for educational purposes, as well as for understanding and quantifying the development of expertise in POCUS [6, 19]. In previous studies, eye-tracking was investigated while participants were looking at prepared sonography images (e.g., trauma sonography, regional anaesthesia, coronary angiogram) and demonstrated that analysis of gaze patterns is even feasible in case of moving POCUS examinations [6, 19–21]. Due to the development of portable ultrasound machines, POCUS is not only reserved to specialists (e.g. cardiologists) anymore. POCUS gains in importance in emergency medicine, even in out-of-hospital scenarios as it may be useful for urgent triage, guidance of procedural interventions, evaluation of clinical symptoms, therapeutic monitoring as well as during cardiopulmonary resuscitation [22, 23]. Although, training in POCUS is recommended for medical students as well as for residents and specialists, the most effective training method for POCUS is unknown. Interpretation of gaze behavior during POCUS examinations, which was demonstrated in this trial to be technically feasible, opens new horizons for educators and trainees as eye-tracking can be used for training, assessment as well as a feedback tool to improve individual’s learning curve.

In this pilot study experts and non-experts had different gaze patterns, although only the duration to first fixation in the AOI was statistically significant (5 s versus 10 s, *p* = 0.033). These findings are comparable with previous studies. Differences in gaze fixation behavior between experts and non-experts were detectable for image interpretation of focused assessment with sonography for trauma, ultrasound-guided regional anaesthesia as well as for coronary angiogram [6, 19–21]. Several studies demonstrated that experts spend less time in identifying anatomy and thus pathologic findings were focused more quickly [6, 24]. In the field of surgery, novices were trained with gaze patterns of experts leading to faster and more accurate performance [8]. Analyzing gaze paths recordings of experts could even be useful for novices trained in POCUS to visualize regions of interests in cases with different pathologies.

This pilot study carries some limitations. First, due to the small sample size interpretation and generalization of the findings of this study should be done with caution. The 95% confidence interval for the percentage of correctly identified AOIs (92.3% to 98.9%) is fully above 90%, which was the acceptable lower bound defined in the study protocol for establishing feasibility. A formal sample size calculation has not been performed for this study, which could have led to insufficient data. However, the low width of the confidence interval demonstrates sufficient power for this feasibility study, though only in a post-hoc manner. Moreover, the non-experts in this study had a median experience of echocardiography of 5 (0.5–7) years and thus cannot be classified as complete novices.

## Conclusion

In conclusion, the findings of this study support the feasibility of eye-tracking during POCUS examinations and suggest that interpretation of gaze patterns has the potential to become a tool for clinical education. For trainees, analysis of gaze behavior may facilitate the evaluation of learning and may provide valuable feedback about ultrasound performance. Future studies are recommended to explore this technology and its relevance for educational purposes of POCUS.

## Supplementary Information

**Additional file 1:** **Supplementary Data 1.** History and clinical symptoms of the scenario. **Supplementary Data 2.** First fixation time in areas of interest.

## Data Availability

The datasets used and analyzed during the current study are available from the corresponding author on reasonable request. Exemplary recordings are available from the corresponding author on request.

## References

[1] Katz TA, Weinberg DD, Fishman CE, Nadkarni V, Tremoulet P, Te Pas AB (2019). Visual attention on a respiratory function monitor during simulated neonatal resuscitation: an eye-tracking study. Arch Dis Child Fetal Neonatal Ed.

[2] Law BHY, Cheung P-Y, Wagner M, van Os S, Zheng B, Schmölzer G. Analysis of neonatal resuscitation using eye tracking: a pilot study. Arch Dis Child Fetal Neonatal Ed. 2018;103:F82–4. 10.1136/archdischild-2017-313114 28824007

[3] Wagner M, Gröpel P, Bibl K, Olischar M, Auerbach MA, Gross IT (2020). Eye-tracking during simulation-based neonatal airway management. Pediatr Res United States.

[4] Tien T, Pucher PH, Sodergren MH, Sriskandarajah K, Yang G-Z, Darzi A (2014). Eye tracking for skills assessment and training: a systematic review. J Surg Res United States.

[5] Brunyé TT, Carney PA, Allison KH, Shapiro LG, Weaver DL, Elmore JG (2014). Eye movements as an index of pathologist visual expertise: a pilot study. PLoS ONE.

[6] Borg LK, Harrison TK, Kou A, Mariano ER, Udani AD, Kim TE, et al. Preliminary Experience Using Eye-Tracking Technology to Differentiate Novice and Expert Image Interpretation for Ultrasound-Guided Regional Anesthesia. J Ultrasound Med. 2018. 10.1002/jum.14334 28777464

[7] Wilson M, McGrath J, Vine S, Brewer J, Defriend D, Masters R (2010). Psychomotor control in a virtual laparoscopic surgery training environment: gaze control parameters differentiate novices from experts. Surg Endosc.

[8] Wilson MR, Vine SJ, Bright E, Masters RSW, Defriend D, McGrath JS (2011). Gaze training enhances laparoscopic technical skill acquisition and multi-tasking performance: a randomized, controlled study. Surg Endosc.

[9] Kelm DJ, Morrow MM, Kennedy CC, Beckman TJ (2020). Feasibility and Utility of an Eye-Tracking Device for Assessing Teachers of Invasive Bedside Procedures. Mayo Clin proceedings Innov Qual outcomes.

[10] Díaz-Gómez JL, Mayo PH, Koenig SJ (2021). Point-of-Care Ultrasonography. N Engl J Med United States.

[11] Moore CL, Copel JA (2011). Point-of-care ultrasonography. N Engl J Med United States.

[12] Ashraf H, Sodergren MH, Merali N, Mylonas G, Singh H, Darzi A (2018). Eye-tracking technology in medical education: A systematic review. Med Teach England.

[13] Harvey A, Vickers JN, Snelgrove R, Scott MF, Morrison S (2014). Expert surgeon’s quiet eye and slowing down: expertise differences in performance and quiet eye duration during identification and dissection of the recurrent laryngeal nerve. Am J Surg.

[14] Vine SJ, Masters RSW, McGrath JS, Bright E, Wilson MR (2012). Cheating experience: Guiding novices to adopt the gaze strategies of experts expedites the learning of technical laparoscopic skills. Surgery.

[15] Soh BP, Reed WM, Poulos A, Brennan PC (2013). E-tutorial improves students’ ability to detect lesions. Radiol Technol.

[16] Sodergren MH, Orihuela-Espina F, Froghi F, Clark J, Teare J, Yang GZ (2011). Value of orientation training in laparoscopic cholecystectomy. Br J Surg.

[17] Wagner M, den Boer MC, Jansen S, Groepel P, Visser R, Witlox RSGM (2022). Video-based reflection on neonatal interventions during COVID-19 using eye-tracking glasses: an observational study. Arch Dis Child Fetal Neonatal Ed.

[18] Johnson PC (2014). Extension of Nakagawa & Schielzeth’s R(2)(GLMM) to random slopes models. Methods Ecol Evol.

[19] Bell CR, Szulewski A, Walker M, McKaigney C, Ross G, Rang L (2021). Differences in Gaze Fixation Location and Duration Between Resident and Fellowship Sonographers Interpreting a Focused Assessment With Sonography in Trauma. AEM Educ Train.

[20] Brunyé TT, Nallamothu BK, Elmore JG (2019). Eye-tracking for assessing medical image interpretation: A pilot feasibility study comparing novice vs expert cardiologists. Perspect Med Educ.

[21] Lee WF, Chenkin J (2021). Exploring Eye-tracking Technology as an Assessment Tool for Point-of-care Ultrasound Training. AEM Educ Train.

[22] Lee L, DeCara JM (2020). Point-of-Care Ultrasound. Curr Cardiol Rep.

[23] Ultrasound Guidelines: Emergency, Point-of-Care and Clinical Ultrasound Guidelines in Medicine. Ann Emerg Med. 2017;69(5):e27–e54. 10.1016/j.annemergmed.2016.08.457. 10.1016/j.annemergmed.2016.08.457 28442101

[24] Harrison TK, Kim TE, Kou A, Shum C, Mariano ER, Howard SK. Feasibility of eye-tracking technology to quantify expertise in ultrasound-guided regional anesthesia. J Anesth. 2016. 10.1007/s00540-016-2157-6 26980475

