# Supplementary material for: Eye-tracking during simulation-based echocardiography: a feasibility study
Source: BMC Med Educ. 2023 Jul 1;23:490. doi: 10.1186/s12909-023-04458-z (PMC10314389; doi:10.1186/s12909-023-04458-z)
Supplement: Supplementary file 1 — Additional file 1: Supplementary Data 1. History and clinical symptoms of the scenario. Supplementary Data 2. First fixation time in areas of interest. [file 12909_2023_4458_MOESM1_ESM.pdf]

Supplementary Data 1: History and clinical symptoms of the scenario

Case 1 (pericardial effusion)
65 years old man was admitted to the emergency department due to general weakness and acute dyspnoea

medical history:
Bronchial Carcinoma

vital parameters:

peripheral oxygen saturation:	91% with supplemental oxygen 15l/min
respiratory rate:	18/min
blood pressure	80/50mmHg
heart rate:	90/min

Case 2 (pulmonary embolism)
35 years old man was admitted to the emergency department due to acute dyspnoea

medial history:
no chronic diseases
long-haul flight 3 days ago

vital parameters:

peripheral oxygen saturation:	85% with supplemental oxygen 15l/min
respiratory rate:	30/min
blood pressure	75/55mmHg
heart rate:	120/min

Case 3 (acute myocardial infarction)
48 years old calls ambulance due to severe chest pain

medial history:
nicotine abuse
hyperlipidemia

vital parameters:

peripheral oxygen saturation:	90%
respiratory rate:	30/min
blood pressure	90/60mmHg
heart rate:	110/min

Case 4 (cardiac myxoma)
66 years old women preanesthetic evaluation (total knee endoprosthesis)

<u>medical history:</u>
no chronic disease

<u>vital parameters:</u>

peripheral oxygen saturation:	97%
respiratory rate:	12/min
blood pressure	120/80mmHg
heart rate:	90/min

Case 5 (bicuspid aortic valve)
13 years old boy preanesthetic evaluation (knee arthroscopy) systolic murmur

<u>medical history:</u>
no chronic disease

<u>vital parameters:</u>

peripheral oxygen saturation:	99%
respiratory rate:	18/min
blood pressure	160/100mmHg
heart rate:	90/min

Case 6 (left ventricular pseudo-aneurysm)
57 years old was admitted to the emergency department due to general weakness and dyspnoea

<u>medial history:</u>
3 weeks ago acute myocardial infarction

<u>vital parameters:</u>

peripheral oxygen saturation:	95%
respiratory rate:	20/min
blood pressure	95/60mmHg
heart rate:	110/min

Supplementary Data 2: First fixation time in areas of interest

time to first fixation in areas of interest	Experts (n=6)	non-experts (n=6)
Case 1 (pericardial effusion)		
apical 4 chamber view	3 (1-15) sec	20 (8-41) sec
parasternal long axis view	5 (2-69) sec	15 (8-42) sec
parasternal short axis view	1 (0-2) sec	2 (1-23) sec
subxiphoidal 4 chamber view	7 (0-43) sec	17 (0-31) sec
Case 2 (pulmonary embolism)		
apical 4 chamber view	7 (1-16) sec	19 (1-32) sec
parasternal long axis view	7 (0-40) sec	19 (10-57) sec
parasternal short axis view	2 (1-30) sec	4 (1-15) sec
subxiphoidal 4 chamber view	3 (0-24) sec	7 (0-12) sec
Case 3 (acute myocardial infarction)		
apical 4 chamber view	5 (1-8) sec	4 (2-25) sec
parasternal long axis view	13 (2-23) sec	13 (3-26) sec
parasternal short axis view	5 (1-20) sec	5 (1-70) sec
subxiphoidal 4 chamber view	5 (0-65) sec	3 (0-13) sec
Case 4 (cardiac myxoma)		
apical 4 chamber view	5 (0-23) sec	8 (1-12) sec
parasternal long axis view	5 (1-27) sec	10 (0-15) sec
parasternal short axis view	4 (2-9) sec	4 (1-20) sec
subxiphoidal 4 chamber view	5 (0-24) sec	0 (0-30) sec
Case 5 (bicuspid aortic valve)		
apical 4 chamber view	10 (1-31) sec	9 (2-22) sec
parasternal long axis view	4 (2-117) sec	21 (8-49) sec
parasternal short axis view	2 (1-8) sec	6 (1-62) sec
subxiphoidal 4 chamber view	7 (0-37) sec	7 (0-63) sec
Case 6 (left ventricular pseudo-aneurysm)		
apical 4 chamber view	7 (2-30) sec	9 (3-24) sec
parasternal long axis view	5 (1-42) sec	19 (1-42) sec
parasternal short axis view	2 (0-9) sec	6 (1-41) sec
subxiphoidal 4 chamber view	2 (0-10) sec	8 (0-60) sec

Data are presented as median (minimum and maximum)
